# Overcoming the Resistance Hurdle: Pharmacokinetic-Pharmacodynamic Target Attainment Analyses for Rezafungin (CD101) against Candida albicans and Candida glabrata

**DOI:** 10.1128/AAC.02614-17

**Published:** 2018-05-25

**Authors:** Justin C. Bader, Elizabeth A. Lakota, Shawn Flanagan, Voon Ong, Taylor Sandison, Christopher M. Rubino, Sujata M. Bhavnani, Paul G. Ambrose

**Affiliations:** a Institute for Clinical Pharmacodynamics, Inc., Schenectady, New York, USA; b Cidara Therapeutics, San Diego, California, USA

**Keywords:** echinocandin, PK-PD target attainment, *Candida glabrata*, *Candida albicans*, *Candida* species

## Abstract

Rezafungin (CD101) is a novel echinocandin antifungal agent with activity against Aspergillus and Candida species, including azole- and echinocandin-resistant isolates. The objective of these analyses was to conduct pharmacokinetic (PK)-pharmacodynamic (PD) target attainment analyses to evaluate single and once-weekly rezafungin dosing to provide dose selection support for future clinical studies. Using a previously developed rezafungin population PK model, Monte Carlo simulations were conducted utilizing the following three intravenous rezafungin regimens: (i) a single 400 mg dose, (ii) 400 mg for week 1 followed by 200 mg weekly for 5 weeks, and (iii) 400 mg weekly for 6 weeks. Percent probabilities of achieving the nonclinical PK-PD targets associated with net fungal stasis and 1-log_10_ CFU reductions from baseline for Candida albicans and Candida glabrata were calculated for each rezafungin regimen. At the MIC_90_ for C. albicans and C. glabrata, a single 400 mg dose of rezafungin achieved probabilities of PK-PD target attainment of ≥90% through week 3 of therapy for all PK-PD targets evaluated. When evaluating the multiple-dose (i.e., weekly) regimens under these conditions, percent probabilities of PK-PD target attainment of 100% were achieved through week 6. Moreover, high (>90%) probabilities of PK-PD target attainment were achieved through week 6 following administration of the weekly regimens at or above the MIC_100_ values for C. albicans and C. glabrata based on contemporary *in vitro* surveillance data. These analyses support the use of single and once-weekly rezafungin regimens for the treatment of patients with candidemia and/or candidiasis due to C. albicans or C. glabrata.

## INTRODUCTION

Clinical practice guidelines recommend the use of echinocandins as first-line therapy for the treatment of candidemia and invasive candidiasis (1). The echinocandins provide clinicians an appealing alternative over more traditional azole and polyene therapies, given their inherently lower likelihood of eliciting drug-drug interactions or drug-related toxicities. Unlike azoles and polyenes, which act by binding to cytochrome P-450 enzymes and sterols, respectively, the echinocandins target 1,3-β-d-glucan synthase, an enzyme complex which is absent from mammalian cells. This enzyme complex is comprised of two subunits: a regulatory GTP-binding protein, Rho1p, and a catalytic component, Fksp, which is encoded by three highly homologous genes, *FKS1*, *FKS2*, and *FKS3* (2, 3). Importantly, hot spot mutations in *FKS1* and *FKS2* have been shown to result in the reduced sensitivity of 1,3-β-d-glucan synthase to echinocandins and elevated echinocandin MIC values across various Candida species (4–6).

Fortunately, incidence rates among Candida isolates with these *fks* mutations are still relatively rare (7); however, reports of their appearance are becoming increasingly frequent (8–17). This trend is especially troubling in light of clinical studies that have associated the presence of *fks* mutations with increased treatment failures (18–21). These factors have collectively raised concerns regarding the lasting utility of current echinocandin therapies and call for the development of new, more efficacious agents to combat resistant Candida isolates.

Rezafungin (CD101) is a novel echinocandin antifungal agent with activity against Aspergillus and Candida spp., including azole- and echinocandin-resistant isolates (22, 23). This structural analog of anidulafungin exhibits a concentration-dependent pattern of fungal killing (24) and a remarkably long half-life in humans of approximately 133 h (25). Similar to the approved echinocandins, rezafungin appears to possess an exceptional margin of safety (25, 26). Rezafungin is an excellent candidate for extended-interval dosing, given its long half-life, apparent wide margin of safety, and concentration-dependent pattern of fungal killing. In fact, a recent evaluation of rezafungin demonstrated that front-loaded regimens achieved greater bacterial killing *in vivo* than more fractionated regimens (27). Accordingly, a once-weekly rezafungin regimen (400 mg intravenously [i.v.] on day 1 followed by 200 mg on day 8 with optional 200 mg doses on days 15 and 22) is being evaluated in an ongoing phase 2 study for the treatment of patients with candidemia and/or invasive candidiasis (ClinicalTrials.gov registration no. NCT02734862). Herein, we describe pharmacokinetic (PK)-pharmacodynamic (PD) target attainment analyses undertaken to evaluate single and once-weekly dosing of rezafungin in order to provide dose selection support for future clinical studies.

## RESULTS

**FIG 1:**
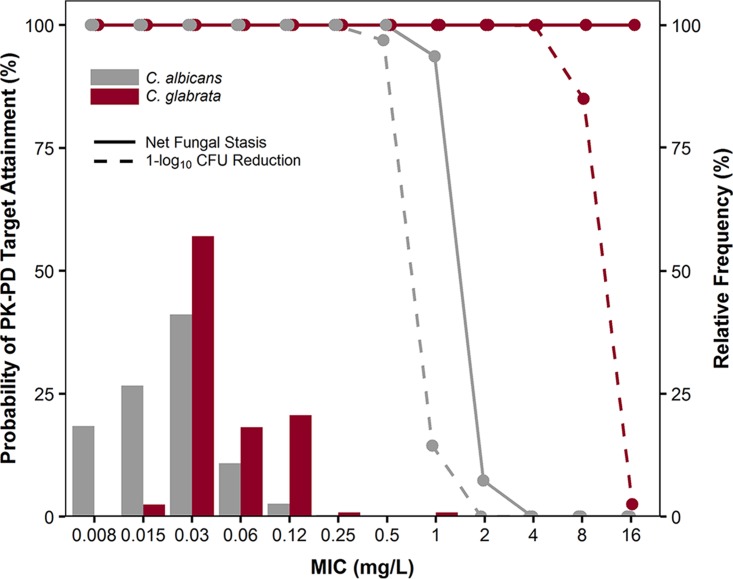
Week 1 percent probabilities of PK-PD target attainment by MIC based on the free-drug AUC_0–168_/MIC ratio targets associated with net fungal stasis and 1-log_10_ CFU reductions from baseline for C. albicans (gray solid and dashed lines, respectively) and C. glabrata (burgundy solid and dashed lines, respectively) among simulated patients administered 400 mg of rezafungin overlaid upon worldwide C. albicans and C. glabrata MIC distributions.

Week 1 percent probabilities of PK-PD target attainment for Candida albicans and Candida glabrata based on their respective area under the concentration-time curve from time zero to 168 h (AUC_0–168_)-to-MIC (AUC_0–168_/MIC) ratio targets associated with net fungal stasis and 1-log_10_ CFU reductions from baseline are shown in Fig. 1.

### PK-PD target attainment analyses for C. albicans.

**FIG 2:**
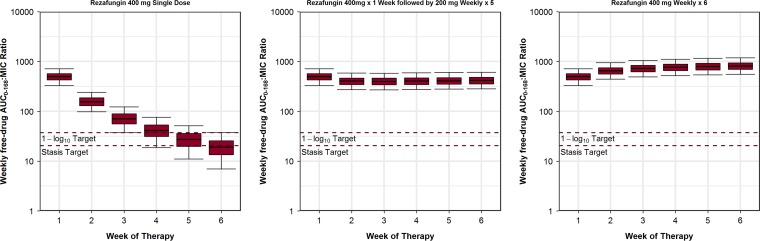
Distributions of free-drug AUC_0–168_/MIC ratios at the MIC_90_ value for C. albicans of 0.06 mg/liter for simulated patients administered the single-dose and weekly rezafungin regimens shown relative to the free-drug AUC_0–168_/MIC ratio targets associated with net fungal stasis and a 1-log_10_ CFU reduction from baseline. Plot whiskers represent the 5th and 95th percentiles of the AUC_0–168_/MIC ratios.

Week 1, 4, and 6 percent probabilities of PK-PD target attainment based on the AUC_0–168_/MIC ratio targets associated with net fungal stasis and 1-log_10_ CFU reductions from baseline for C. albicans for simulated patients administered rezafungin regimen are presented in Table 1. The distributions of free-drug AUC_0–168_/MIC ratios over weeks 1 to 6 based on the C. albicans MIC_90_, 0.06 mg/liter, for simulated patients administered each of the rezafungin regimens are presented in Fig. 2.

**TABLE 1:**
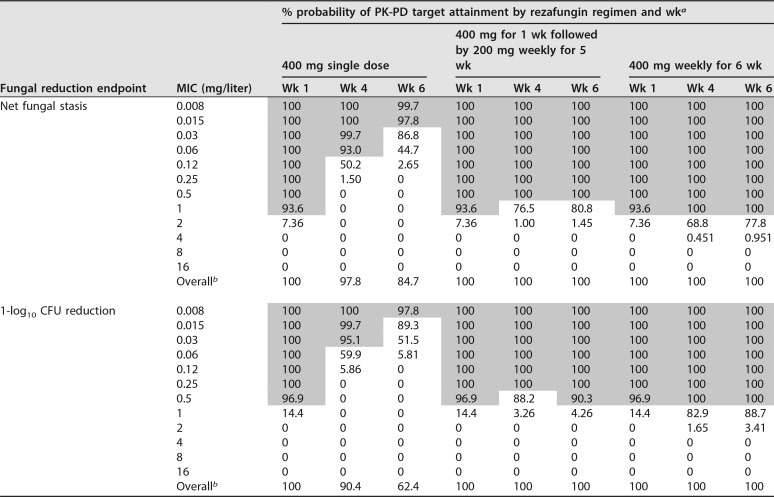
For C. albicans, percent probabilities of PK-PD target attainment by MIC and for simulated patients randomly assigned MIC values based on nonclinical AUC_0–168_/MIC ratio targets associated with net fungal stasis and a 1-log_10_ CFU reduction from baseline following administration of single-dose and weekly rezafungin regimens

^*a*^ Shaded cells indicate percent probabilities of PK-PD target attainment of ≥90%.

^*b*^ Simulated patients were randomly assigned MIC values based on the C. albicans *in vitro* surveillance data presented in Table 3.

At the C. albicans MIC_90_ of 0.06 mg/liter, percent probabilities of PK-PD target attainment based on the AUC_0–168_/MIC ratio target associated with net fungal stasis for C. albicans were ≥90% through weeks 4 and 6 following administration of the single-dose and once-weekly regimens, respectively. For the analyses based on the 1-log_10_ CFU reduction endpoint, these values were weeks 3 and 6, respectively. When based on the net fungal stasis endpoint, the highest MIC values at which percent probabilities of PK-PD target attainment of ≥90% were achieved through week 6 following administration of the 200 and 400 mg weekly regimens were 0.5 and 1 mg/liter, respectively. The highest MIC values at which percent probabilities of PK-PD target attainment of ≥90% were achieved based on the 1-log_10_ CFU reduction endpoint were 0.25 and 0.5 mg/liter, respectively.

Results for the PK-PD target attainment analyses based on patient exposures generated with inflated interindividual variability demonstrated either no change or a 1-dilution decrease in the MIC at which percent probabilities of PK-PD target attainment of ≥90% were achieved relative to the above-described results (see Table S1 and Fig. S1 and S2 in the supplemental material).

### PK-PD target attainment analyses for C. glabrata.

**FIG 3:**
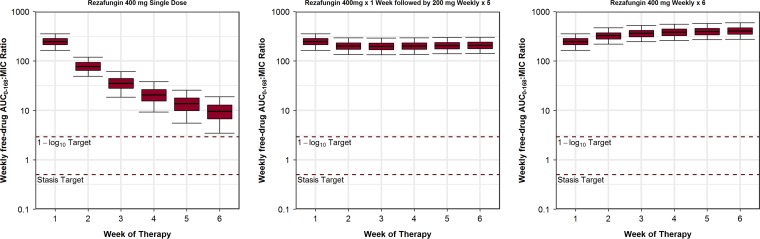
Distributions of free-drug AUC_0–168_/MIC ratios at the MIC_90_ value for C. glabrata of 0.12 mg/liter for simulated patients administered the single-dose and weekly rezafungin regimens shown relative to the free-drug AUC_0–168_/MIC ratio targets associated with net fungal stasis and a 1-log_10_ CFU reduction from baseline. Plot whiskers represent the 5th and 95th percentiles of the AUC_0–168_/MIC ratios.

Week 1, 4, and 6 percent probabilities of PK-PD target attainment based on the AUC_0–168_/MIC ratio targets associated with net fungal stasis and 1-log_10_ CFU reductions from baseline for C. glabrata for simulated patients administered rezafungin regimens are presented in Table 2. The distributions of the free-drug AUC_0–168_/MIC ratios over weeks 1 to 6 based on the C. glabrata MIC_90_, 0.12 mg/liter, for simulated patients administered each of the rezafungin regimens are presented in Fig. 3.

**TABLE 2:**
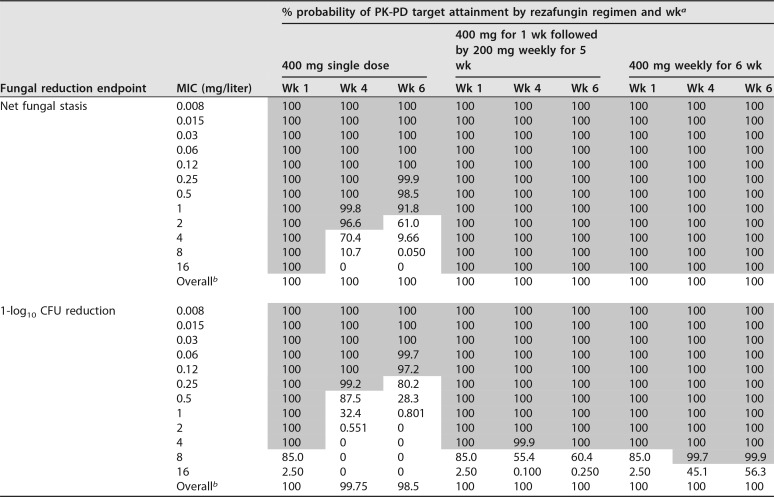
For C. glabrata, percent probabilities of PK-PD target attainment by MIC and for simulated patients randomly assigned MIC values based on nonclinical AUC_0–168_/MIC ratio targets associated with net fungal stasis and a 1-log_10_ CFU reduction from baseline following administration of single-dose and weekly rezafungin regimens

^*a*^ Shaded cells indicate percent probabilities of PK-PD target attainment of ≥90%.

^*b*^ Simulated patients were randomly assigned MIC values based on the C. glabrata *in vitro* surveillance data presented in Table 3.

At the C. glabrata MIC_90_ of 0.12 mg/liter, percent probabilities of PK-PD target attainment were ≥90% through week 6 for all regimens regardless of the fungal reduction endpoint evaluated. When based on the net fungal stasis and 1-log_10_ CFU reduction endpoints, the highest MIC values at which percent probabilities of PK-PD target attainment of ≥90% were achieved through week 6 following administration of the once-weekly regimens were ≥16 mg/liter and 4 mg/liter, respectively.

Results for the PK-PD target attainment analyses based on patient exposures generated with inflated interindividual variability demonstrated either no change or a 1-dilution decrease in the MIC at which percent probabilities of PK-PD target attainment of ≥90% were achieved relative to the above-described results (Table S2 and Fig. S1 and S3).

## DISCUSSION

The objective of these analyses was to carry out PK-PD target attainment analyses evaluating single-dose and once-weekly rezafungin regimens to provide dose selection support for future clinical studies. These analyses were carried out using a population PK model, nonclinical PK-PD targets for efficacy, and *in vitro* surveillance data through Monte Carlo simulation (23, 28, 29).

These PK-PD target attainment analyses were based on the AUC_0–168_/MIC ratio targets associated with net fungal stasis for C. albicans and C. glabrata, given that prior analyses established concordance between this preclinical endpoint and mycological efficacy in micafungin-treated patients with candidemia and invasive candidiasis (30). In order to further evaluate these regimens, PK-PD target attainment analyses were also conducted utilizing AUC_0–168_/MIC ratio targets associated with 1-log_10_ CFU reductions from baseline.

At the MIC_90_ for C. albicans and C. glabrata, the single-dose rezafungin regimen (rezafungin 400 mg) achieved percent probabilities of PK-PD target attainment of ≥90% through week 3 of therapy, regardless of the pathogen or fungal reduction endpoint evaluated, a time frame that is consistent with the average duration of therapy (approximately 14 days) reported for echinocandin-treated patients with candidemia and/or invasive candidiasis enrolled in clinical studies (31–41). Both once-weekly rezafungin regimens achieved percent probabilities of PK-PD target attainment of 100% through week 6, regardless of the pathogen-specific MIC_90_ value or the fungal reduction endpoint evaluated. These single-dose and once-weekly regimens offer several advantages over the traditional daily dosing of other echinocandins, such as improved patient compliance and the reduced use of resources associated with patient monitoring and drug administration.

Moreover, favorable probabilities of PK-PD target attainment following administration of the once-weekly regimens were achieved above the MIC_90_ values for C. albicans and C. glabrata. For the evaluations based on C. albicans, percent probabilities of PK-PD target attainment of ≥90% were achieved through week 6 following administration of the 200 mg and 400 mg weekly regimens at MIC values of 0.5 and 1 mg/liter, respectively (1- and 2-dilution shifts above the MIC_100_, respectively), when based on the net fungal stasis endpoint, and 0.25 and 0.5 mg/liter, respectively (0- and 1-dilution shifts above the MIC_100_, respectively), when based on the 1-log_10_ CFU reduction endpoint. For the evaluations based on C. glabrata, both regimens achieved percent probabilities of PK-PD target attainment of ≥90% through week 6 at MIC values of ≥16 and 4 mg/liter, respectively (4- and 2-dilution shifts above the MIC_100_, respectively), when based on the net fungal stasis and 1-log_10_ CFU reduction endpoints.

However, as previously stated, concerns are growing regarding the threat posed by Candida spp. with mutant *fks* genes, the prevalence of which is greatest among C. glabrata isolates. Median spontaneous mutation frequencies for rezafungin across various Candida spp. have been shown to be similar to those for anidulafungin and caspofungin (1.35 × 10^−8^ to 3.86 × 10^−9^, 1.59 × 10^−7^ to <3.86 × 10^−9^, and 3.45 × 10^−7^ to <3.86 × 10^−9^, respectively) (42). Likewise, rezafungin MIC shifts for the spontaneous *fks* mutants relative to the MIC values for wild-type isolates were comparable to those for anidulafungin and caspofungin among mutant C. albicans (3-, 4-, and 1-dilution shifts, respectively) and C. glabrata (1- to 5-, 2- to 6-, and 0- to 6-dilution shifts, respectively) isolates. The highest rezafungin MIC values across these *fks* mutants were 0.25 and 2 mg/liter for C. albicans and C. glabrata, respectively. Therefore, the percent probabilities of PK-PD target attainment achieved at these MIC values are of particular interest.

The results for the single-dose regimen demonstrated that percent probabilities of PK-PD target attainment of ≥90% were achieved only through weeks 2 and 1 for C. albicans at an MIC of 0.25 mg/liter and through weeks 4 and 2 for C. glabrata at an MIC of 2 mg/liter, when evaluated on the basis of their respective net fungal stasis and 1-log_10_ CFU reduction endpoints, respectively. However, both of these mutant MIC values are at or below the highest MIC values at which the weekly regimens were able to achieve percent probabilities of PK-PD target attainment of ≥90% through week 6, regardless of the fungal reduction endpoint evaluated. These results suggest that once-weekly rezafungin regimens are able to achieve exposures associated with efficacy even against some *fks* mutant C. albicans and C. glabrata isolates.

As described above, percent probabilities of PK-PD target attainment were interpreted relative to the MIC_100_ values for C. albicans and C. glabrata (0.25 and 1 mg/liter, respectively) based on a contemporary collection of Candida isolates (23). While this collection represents the most robust *in vitro* surveillance data for rezafungin currently available, rezafungin MIC_100_ values as high as 0.5 mg/liter and 4 mg/liter for C. albicans and C. glabrata, respectively (43, 44), have been reported. These data suggest that the true rezafungin MIC_100_ values for these pathogens may be underestimated and that MIC values of 0.25 mg/liter for C. albicans and 1 mg/liter for C. glabrata may instead represent the MIC_99_ and MIC_96_, respectively. However, regardless of the true MIC_100_ values for these organisms, high percent probabilities of PK-PD target attainment were evident on the basis of the observed MIC values for all isolates from recent *in vitro* surveillance data (23). Thus, rezafungin regimens based on MIC values for isolates most likely to be encountered clinically are expected to achieve exposures associated with efficacy for the majority of patients.

An additional limitation of these analyses was the allometric weight relationship incorporated into the population PK model used to generate exposures for simulated patients with weights outside those included in the model analysis data set. However, by utilizing a wide range of weights, a wider range of simulated exposures was generated, thus mimicking a distribution of patient exposures that would more likely be observed clinically than those based on the narrow range of weights captured in the homogeneous data set used to develop the rezafungin population PK model. Additionally, the use of allometric scaling as described herein is strongly supported to characterize the impact of weight on drug disposition (45).

In summary, these analyses support the use of single and weekly rezafungin regimens for the treatment of patients with candidemia and/or candidiasis due to C. albicans and C. glabrata. Moreover, the results presented suggest that weekly regimens can achieve exposures associated with efficacy against some *fks* mutant Candida isolates. These single and weekly regimens present the opportunity to deliver drug exposures in a PK-PD-optimized manner, improve patient compliance, and reduce the use of resources associated with patient monitoring and drug administration.

## MATERIALS AND METHODS

A previously developed rezafungin population PK model (28) and nonclinical PK-PD targets for efficacy against C. albicans and C. glabrata (29) were utilized through Monte Carlo simulation to evaluate percent probabilities of PK-PD target attainment by MIC and overall. The results of these analyses were interpreted in the context of C. albicans and C. glabrata MIC distributions for rezafungin (23).

### Population pharmacokinetic model.

Development of the population PK model utilized to characterize the disposition of rezafungin in plasma was extensively described elsewhere (28). In brief, data for this model were obtained from two phase 1 studies (25). The first of these was a single-ascending-dose study evaluating rezafungin i.v. doses ranging from 50 mg to 400 mg. The second was a multiple-ascending-dose study evaluating rezafungin i.v. doses ranging from 100 mg to 400 mg once weekly for two to three doses. These data were best described using a four-compartment model with zero-order drug input and first-order, linear elimination. All parameters in the model were scaled to subject body weight using standard allometric coefficients (powers of 0.75 and 1 for the clearance and volume terms, respectively).

### Nonclinical pharmacokinetic-pharmacodynamic targets for efficacy.

Given that rezafungin exhibits a concentration-dependent pattern of fungal killing (24), nonclinical median free-drug AUC_0–168_/MIC ratio targets for efficacy associated with net fungal stasis and 1-log_10_ CFU reductions from baseline were utilized for these analyses (29). These targets were obtained from neutropenic murine disseminated candidiasis models and were 20.46 and 37.24, respectively, for C. albicans and 0.50 and 2.94, respectively, for C. glabrata.

### Rezafungin *in vitro* activity.

The rezafungin MIC distributions for C. albicans and C. glabrata isolates collected worldwide during the 2015 SENTRY Surveillance Program (23) that were used to interpret the PK-PD target attainment results and calculate overall percent probabilities of PK-PD target attainment are summarized in Table 3. Among the 304 C. albicans and 121 C. glabrata isolates collected, MIC_90_ values were 0.06 and 0.12 mg/liter, respectively.

**TABLE 3:**
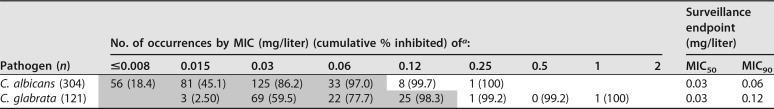
Rezafungin MIC distributions for C. albicans and C. glabrata based on isolates collected worldwide

^*a*^ Based on data for clinical C. albicans and C. glabrata isolates described in reference 23. Shaded cells represent the MIC values up to and including the MIC_90_, and values in parentheses represent the cumulative percentage of isolates inhibited.

### PK-PD target attainment analyses.

Using the above-described population PK model and a protein-binding estimate of 97.4% (46), a Monte Carlo simulation was conducted utilizing the mrgsolve package in R (version 3.3.1). In this simulation, free-drug plasma concentration-time profiles over 6 weeks were generated for 2,000 simulated patients following administration of each of the following i.v. rezafungin regimens: a single 400 mg dose, 400 mg for week 1 followed by 200 mg weekly for 5 weeks, and 400 mg weekly for 6 weeks. Given that weight was a covariate in the population PK model, each subject was randomly assigned a weight from a data set of demographic information collected from patients with pneumonia and skin infections (Institute for Clinical Pharmacodynamics, Inc., data on file). The median weight in the data set was 78 kg, with a range of 33.8 kg to 227 kg. Weekly free-drug plasma AUC values (AUC_0–168_) were calculated for each subject following administration of rezafungin through numeric integration of free-drug plasma concentration-time profiles for each week from weeks 1 through 6.

The free-drug plasma AUC_0–168_/MIC ratio was then calculated for each simulated subject using individual MIC values ranging from 0.03 mg/liter to 16 mg/liter and by randomly assigning MIC values on the basis of the rezafungin MIC distributions for C. albicans and C. glabrata shown in Table 3. Percent probabilities of PK-PD target attainment by week were calculated for each rezafungin regimen.

Given that the rezafungin population PK model was developed exclusively using healthy volunteer data, it is possible that interindividual variability is underestimated relative to what would be observed in patients. Therefore, additional PK-PD target attainment analyses for which interindividual variability was inflated on all model parameter estimates were performed. Coefficients of variation were increased to 40% for all interindividual variability terms below this value (47, 48, 49).

## Supplementary Material

Supplemental material
