# Supplementary material for: Overcoming the Resistance Hurdle: Pharmacokinetic-Pharmacodynamic Target Attainment Analyses for Rezafungin (CD101) against Candida albicans and Candida glabrata
Source: Antimicrob Agents Chemother. 2018 May 25;62(6):e02614-17. doi: 10.1128/AAC.02614-17 (PMC5971579; doi:10.1128/AAC.02614-17)
Supplement: Supplemental material [file supp_62_6_e02614-17__index.html]

Supplemental material 

# Overcoming the Resistance Hurdle: Pharmacokinetic-Pharmacodynamic Target Attainment Analyses for Rezafungin (CD101) against Candida albicans and Candida glabrata

## Supplemental material

- Supplemental file 1 -

  Supplemental Tables S1 and S2 and Figures S1 to S3

  PDF, 455K
