# Supplementary material for: Overcoming the Resistance Hurdle: Pharmacokinetic-Pharmacodynamic Target Attainment Analyses for Rezafungin (CD101) against Candida albicans and Candida glabrata
Source: Antimicrob Agents Chemother. 2018 May 25;62(6):e02614-17. doi: 10.1128/AAC.02614-17 (PMC5971579; doi:10.1128/AAC.02614-17)

Supplemental Table 1. For *C. albicans*: Percent probabilities of PK-PD target attainment by MIC and for simulated patients with inflated interindividual variability randomly assigned MIC values based on non-clinical AUC_{0-168}:MIC ratio targets associated with net fungal stasis and a 1-log_{10} CFU reduction from baseline following administration of single-dose and weekly rezafungin regimens

Fungal reduction endpoint	MIC (mg/L)	Percent probabilities of PK-PD target attainment by rezafungin regimens and Week[a]								
		400 mg Single Dose			400 mg x 1 Week followed by 200 mg Weekly x 5			400 mg Weekly x 6		
		Week 1	Week 4	Week 6	Week 1	Week 4	Week 6	Week 1	Week 4	Week 6
Net fungal stasis	0.008	100	99.2	94.8	100	100	100	100	100	100
	0.015	100	97.6	88.1	100	100	100	100	100	100
	0.03	100	91.6	72.7	100	100	100	100	100	100
	0.06	100	76.2	48.4	100	100	100	100	100	100
	0.12	100	51.0	20.0	100	100	100	100	100	100
	0.25	100	17.2	3.66	100	100	100	100	100	100
	0.5	99.9	1.95	0.150	99.9	97.1	97.5	99.9	100	100
	1	84.5	0	0	84.5	64.4	66.8	84.5	96.7	97.1
	2	9.72	0	0	9.72	7.26	9.61	9.72	59.9	64.9
	4	0	0	0	0	0	0.100	0	4.61	7.81
	8	0	0	0	0	0	0	0	0	0.050
	16	0	0	0	0	0	0	0	0	0
	Overall[b]	100	91.7	76.5	100	100	100	100	100	100
1-log_{10} CFU reduction	0.008	100	97.6	88.6	100	100	100	100	100	100
	0.015	100	92.7	75.1	100	100	100	100	100	100
	0.03	100	80.3	51.7	100	100	100	100	100	100
	0.06	100	55.8	23.5	100	100	100	100	100	100
	0.12	100	23.3	5.06	100	100	100	100	100	100
	0.25	99.9	2.80	0.351	99.9	98.5	98.7	99.9	100	100
	0.5	90.2	0	0	90.2	71.9	74.6	90.2	98.1	98.5
	1	16.3	0	0	16.3	11.6	14.6	16.3	68.5	72.7
	2	0.100	0	0	0.100	0.050	0.250	0.100	7.66	12.5
	4	0	0	0	0	0	0	0	0	0.250
	8	0	0	0	0	0	0	0	0	0
	16	0	0	0	0	0	0	0	0	0
	Overall[b]	100	82.2	58.5	100	100	100	100	100	100

a. Shaded cells indicate PK-PD target attainment values ≥90%.
b. Simulated patients were randomly assigned MIC values based on the *C. albicans in vitro* surveillance data presented in Table 3.

Supplemental Table 2. For *C. glabrata*: Percent probabilities of PK-PD target attainment by MIC and for simulated patients with inflated interindividual variability randomly assigned MIC values based on non-clinical $AUC_{0\text{-}168}$:MIC ratio targets associated with net fungal stasis and a 1-$\log_{10}$ CFU reduction from baseline following administration of single-dose and weekly rezafungin regimens

Fungal reduction endpoint	MIC (mg/L)	Percent probabilities of PK-PD target attainment by rezafungin regimens and Week[a]								
		400 mg Single Dose			400 mg x 1 Week followed by 200 mg Weekly x 5			400 mg Weekly x 6		
		Week 1	Week 4	Week 6	Week 1	Week 4	Week 6	Week 1	Week 4	Week 6
Net fungal stasis	0.008	100	100	99.9	100	100	100	100	100	100
	0.015	100	100	99.8	100	100	100	100	100	100
	0.03	100	100	99.5	100	100	100	100	100	100
	0.06	100	99.9	99.0	100	100	100	100	100	100
	0.12	100	99.8	98.0	100	100	100	100	100	100
	0.25	100	99.6	96.0	100	100	100	100	100	100
	0.5	100	98.3	91.7	100	100	100	100	100	100
	1	100	94.0	78.6	100	100	100	100	100	100
	2	100	82.3	56.2	100	100	100	100	100	100
	4	100	60.2	27.9	100	100	100	100	100	100
	8	100	28.3	7.26	100	100	100	100	100	100
	16	100	5.31	0.601	100	99.5	99.5	100	100	100
	Overall[b]	100	99.9	99.0	100	100	100	100	100	100
1-$\log_{10}$ CFU reduction	0.008	100	100	99.4	100	100	100	100	100	100
	0.015	100	99.9	98.6	100	100	100	100	100	100
	0.03	100	99.8	97.2	100	100	100	100	100	100
	0.06	100	99.2	94.6	100	100	100	100	100	100
	0.12	100	96.7	85.5	100	100	100	100	100	100
	0.25	100	89.0	66.7	100	100	100	100	100	100
	0.5	100	71.7	40.7	100	100	100	100	100	100
	1	100	42.8	14.1	100	100	100	100	100	100
	2	100	12.6	2.35	100	100	100	100	100	100
	4	99.7	0.851	0.050	99.7	94.7	95.1	99.7	100	100
	8	73.5	0	0	73.5	50.4	54.4	73.5	94.2	94.8
	16	4.01	0	0	4.01	3.01	4.81	4.01	44.3	51.8
	Overall[b]	100	98.7	93.4	100	100	100	100	100	100

a. Shaded cells indicate PK-PD target attainment values ≥90%.

b. Simulated patients were randomly assigned MIC values based on the *C. glabrata in vitro* surveillance data presented in Table 3.

Supplemental Figure 1. Week 1 percent probabilities of PK-PD target attainment by MIC based on the free-drug $AUC_{0\text{-}168}$:MIC ratio targets associated with net fungal stasis and 1-$\log_{10}$ CFU reductions from baseline for *C. albicans* (grey solid and dashed lines, respectively) and *C. glabrata* (burgundy solid and dashed lines, respectively) among simulated patients with inflated interindividual variability administered 400 mg of rezafungin overlaid upon worldwide *C. albicans* and *C. glabrata* MIC distributions

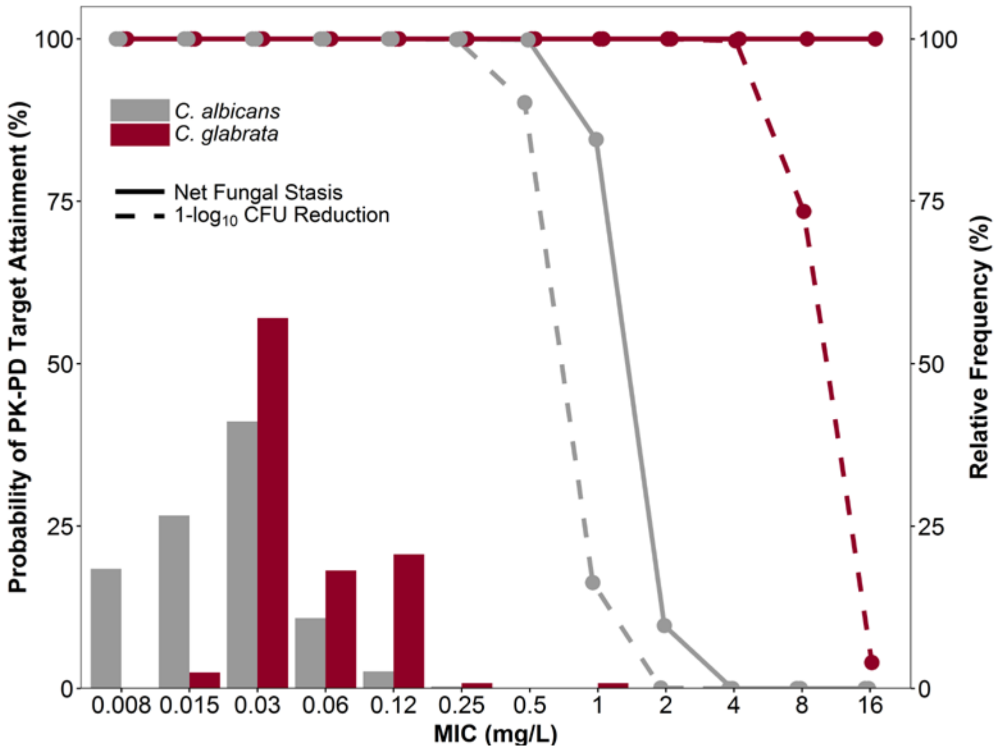

Supplemental Figure 2. Distributions of free-drug AUC_{0-168}:MIC ratios at the MIC_{90} value for *C. albicans* of 0.06 mg/L for simulated patients with inflated interindividual variability administered single-dose and weekly rezafungin regimens shown relative to free-drug AUC_{0-168}:MIC ratio targets associated with net fungal stasis and a 1-log_{10} CFU reduction from baseline. Plot whiskers represent the 5th and 95th percentiles of AUC_{0-168}:MIC ratios.

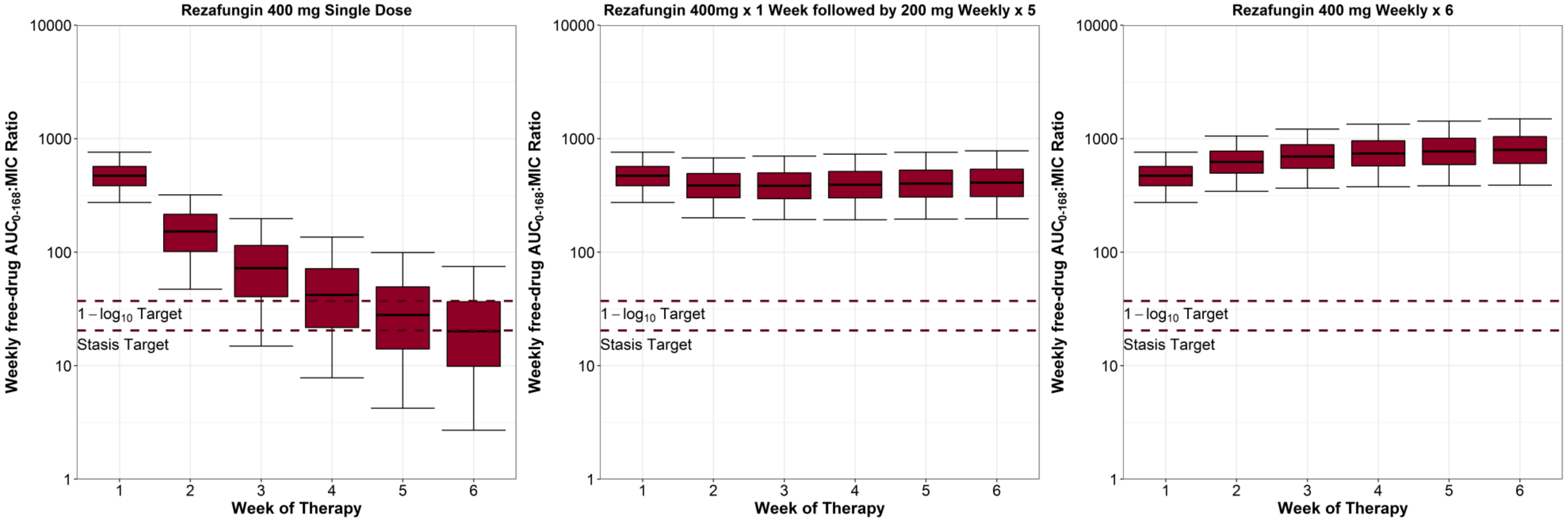

Supplemental Figure 3. Distributions of free-drug $AUC_{0\text{-}168}$:MIC ratios at the MIC_{90} value for *C. glabrata* of 0.12 mg/L for simulated patients with inflated interindividual variability administered single-dose and weekly rezafungin regimens shown relative to the free-drug $AUC_{0\text{-}168}$:MIC ratio targets associated with net fungal stasis and a 1-log_{10} CFU reduction from baseline. Plot whiskers represent the 5th and 95th percentiles of $AUC_{0\text{-}168}$:MIC ratios.

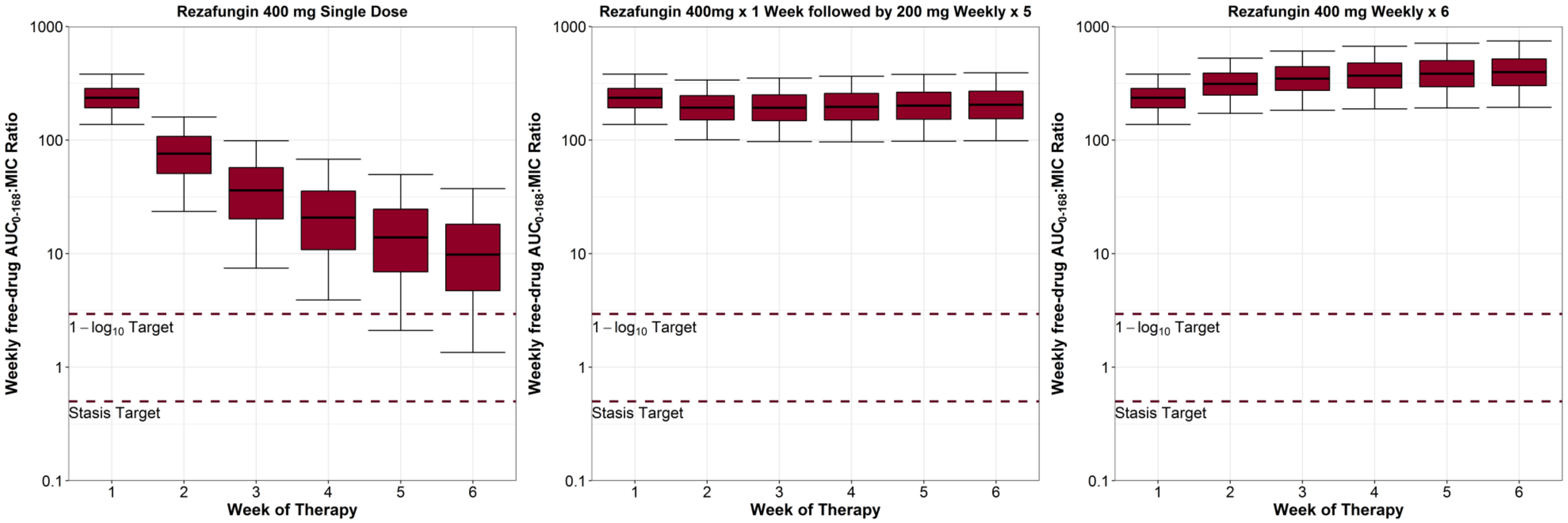

Supplement: Supplemental material [file AAC.02614-17_zac006187178s1.pdf]
